# Barriers and enablers to menu planning guideline implementation in Australian childcare centres and the role of government support services

**DOI:** 10.1017/S1368980022001343

**Published:** 2022-06-01

**Authors:** Audrey Elford, Alison Spence, Amy Wakem, Karen J Campbell, Penelope Love

**Affiliations:** 1 Institute for Physical Activity and Nutrition (IPAN), School of Exercise and Nutrition Sciences (SENS), Deakin University Geelong Waurn Ponds Campus, 75 Pigdons Road, Geelong, VIC 3216, Australia; 2 Healthy Eating Advisory Service, Nutrition Australia, VIC, Australia

**Keywords:** Early childhood, Childcare, Nutrition, Menu compliance

## Abstract

**Objective:**

To explore government support service access, perceived barriers/enablers to menu planning and menu compliance in long day care (LDC) centres in Victoria, Australia, where the Victorian Government-funded Healthy Eating Advisory Service (HEAS) is available to provide free LDC menu planning support.

**Design:**

This is a cross-sectional study design. Data were collected from online surveys with the option of uploading 2 weeks of menus and recipes. Menu compliance was scored for quantity, quality and variety. Barriers/enablers to menu planning guideline implementation were determined using the Theoretical Domains Framework (TDF). Independent *t* tests, one-way ANOVA and chi-square tests assessed relationships between characteristics, barriers/enablers and menu scores.

**Setting:**

Eighty-nine LDC centres that prepare food onsite.

**Participants:**

LDC staff responsible for menu planning (*n* 89) and menus from eighteen centres.

**Results:**

Fifty-five per cent of centres had accessed HEAS. Of eighteen provided menus, only one menu was compliant with menu planning guidelines. HEAS access was associated with higher average scores in four of seven TDF domains, namely knowledge/awareness, skills/role, reinforcement/influence and optimism/intent. There were no correlations between menu score and barriers/enablers; however, menu quality scores were higher for those accessing HEAS.

**Conclusions:**

Childcare-specific government support services may be an important public health nutrition strategy and may improve menu planning guideline implementation as well as menu quality; however, this does not necessarily translate into menu compliance. Research should confirm these findings in larger studies to ascertain uptake of these services. Public health efforts should focus on exploring barriers and enablers to uptake of government support services to increase reach and acceptability.

The uptake of childcare in high-income countries is growing, with approximately 40 % of children aged 3–4 years in the USA attending childcare^(1)^, 64 % in the United Kingdom and 90 % in New Zealand^(2,3)^. Around 50 % of children in Australia aged 2–5 years attend childcare in the form of long day care (LDC). LDC can be defined as centre-based care for children aged 0–6 years, operating at least 8 h/d, 48 weeks/year^(4)^. Children spend a significant amount of time in childcare; for example, Australian children under 5 years of age spend an average of 31 h/week (equating to more than four 7-h days) in childcare, and children in the United Kingdom spend an average of 21 h in childcare (equating to three 7-h days)^(2,5)^. Many LDC childcare services provide children with a minimum of one main meal and two snacks which is prepared onsite and constitute approximately half of their nutritional intake for the day^(6)^. This makes childcare an important setting for public health nutrition interventions.

Early childhood is a critical life stage for focusing on nutrition and obesity prevention strategies, as food preferences are formed during this time and carry through to adolescence and adulthood, influencing the risk of developing obesity and non-communicable diseases^(7,8)^. Research on food consumption patterns in children aged 2–5 years indicates underconsumption of vegetables and overconsumption of discretionary foods (a term used in Australia to define foods that are not core five food group items, generally high in sugar, fat or salt, such as chocolate, jelly, commercially made biscuits, ice cream, soft drinks, deep-fried foods and takeaway foods)^(9)^.

Research on food provision in LDC settings indicates that foods served to children aged 2–5 years do not meet nutrition recommendations, with an overarching theme of underprovision of vegetables, wholegrains and meat/alternatives and overprovision of discretionary foods and foods high in fat and Na^(10–12)^. Some identified barriers to the planning and preparation of healthy menus include insufficient knowledge and skills on how to implement nutritional guidelines, guideline complexities influencing interpretability for childcare food service, costs of providing a healthy menu, children’s food preferences, lack of government-funded support and food wastage^(13,14)^. Several implementation trials have sought to address menu compliance barriers, with mixed results, and many require high-intensity support that is not sustainable in the long term^(15,16)^.

In the USA and Australia, LDC-specific government-funded nutrition support services have been created as scalable, longer-term solutions to overcome barriers to menu planning and improve food provision in LDC services^(17–19)^. For example, the US Child and Adult Care Food Program (CACFP) reimburses eligible low-income childcare centres for providing children with nutritious meals in line with childcare-specific guidelines^(17)^. In Australia, all childcare services are registered through the Australian Children’s Education and Care Quality Authority (ACECQA)^(20)^ with support provided at a state level which may differ in the type of support provided and whether a free resource, paid service or via membership. In the state of Victoria, Nutrition Australia is funded by the Victorian Government to deliver the Healthy Eating Advisory Service (HEAS). It provides a range of resources and support at no cost to centres through the LDC-specific menu planning guidelines, online resources (example menus and menu planning tips), face-to-face and online training for cooks and educators, telephone support, and a free online menu planning review tool, the Victorian Government’s FoodChecker^(19,21)^.

In considering the effectiveness of these programmes on menu compliance, studies show mixed results^(22,23)^. In the USA, limited awareness about the CACFP and its eligibility criteria have been reported as barriers to participation^(24)^; however, no research has been conducted to explore the barriers and enablers to the planning and provision of compliant menus in centres that access CACFP. In Australia, research on the uptake of a free government-funded service, such as HEAS, and its potential influence on menu planning and compliance is lacking. There is also limited research exploring barriers and enablers to planning compliant menus experienced by centres that do access free childcare-specific menu planning support.

Therefore, this study aimed to explore LDC services in the state of Victoria with access to the free HEAS service regarding: (1) staff characteristics and engagement with HEAS and other sources of support, and adherence to menu planning guidelines; (2) staff perceptions of the barriers and enablers to menu planning, and relationships to menu compliance; and (3) relationships between barriers and enablers to menu planning, and engagement with support services.

## Methods

### Recruitment

Data were collected between 2018 and 2019 in Victoria, Australia. All Victorian childcare centres (*n* 1442) listed on the Australian Government childcare website^(25)^ in February 2018 were invited to participate via email to centre directors. Individual participant consent was obtained via a consent form emailed back to the principal researcher, after which a link to a confidential survey was returned for completion. Eligibility criteria included centres operating for at least 8 h/d and 48 weeks/annum, and providing food onsite. Of the responses received (*n* 159), nineteen were ineligible, two were eligible but did not provide consent, 138 centres provided consent, and eighty-nine centres completed the survey. As there is no publicly available information identifying which services provided food onsite, it was not possible to describe the number of eligible services. This limitation was also described by Gerritsen *et al*.^(11)^ who, in a similar study, were unable to access nationally descriptive data for LDC.

The person responsible for menu planning in the centre was invited to complete a 46-item online survey hosted on Qualtrics platform (Qualtrics, Provo, UT), with an additional option to provide the current centre menu and recipes for review. The survey was developed utilising relevant components of published Early Childhood Education and Care (ECEC) surveys to capture data regarding centre characteristics, support strategies, barriers and enablers to menu planning^(11,26,27)^. Face validity of survey items was checked by nutrition and early childhood and education experts resulting in the rewording/removal of some items. The survey was then pilot-tested by seven childcare services based outside Victoria which informed the final selection of items.

### Measures

#### Centre characteristics

Data on centre demographics, child attendance and menu review details were collected. Centres were categorised according to locality using postcodes (rural/metropolitan)^(28)^ and centre ownership – private (privately run) or community (sponsored by government/church or run by a committee of management) – by individually checking details for each participating centre listed on the government childcare website, cross-checked by a second researcher. Socio-Economic Indexes for Areas (SEIFA) deciles were determined for each centre (one representing most disadvantaged to ten least disadvantaged)^(29)^. SEIFA deciles were further categorised into low (deciles 1–3), medium (deciles 4–7) and high (deciles 8–10)^(30)^.

#### Person responsible for food provision tasks

Data regarding respondent’s role (cook/manager director/educator) and responsibilities pertaining to food provision including budgeting, purchasing, preparing and serving food were collected via predefined answer options.

#### Support services accessed for menu planning

Details on nutrition training to support food provision roles and access to various support for menu planning including HEAS, Victorian Achievement Program (a Victorian Government-funded programme that recognises healthy early childhood services in six areas, including healthy eating)^(31)^ websites, social media and newsletters were obtained using fifteen purpose-designed, predefined questions.

#### Menu compliance and the menu scoring tool

Centres were invited to provide full menus and recipes for two continuous weeks. Data were entered into the Victorian Government’s menu review tool, FoodChecker, to assess for compliance against the Victorian menu planning guidelines for LDC^(19)^. Where menu data were missing, centres were contacted to obtain exact quantities for analysis. Only centres providing all information over the 2-week period were included in the menu analysis. The Victorian LDC menu planning guidelines include recommendations for quantities, quality and variety^(19)^. To capture this, a menu scoring tool was used to assign scores for: quantity (defined as the amount of each food group served/d); quality (defined as quality within food groups, e.g. wholegrains, discretionary foods and type of fats utilised) and variety (defined as the variety of vegetables, fruit and meat served over a 2-week period).

Supplemental Table 1 provides details of the menu scoring tool, modified to the Victorian LDC menu planning guidelines, which was based on a tool utilised in previous research, but which has not been validated^(11,19,32)^. The previously utilised menu scoring tool combined points for each week that menus were compliant; however, the modified tool allocated a point for each day that menus were compliant. This modification aimed to make the menu scoring tool more sensitive to subtle differences. Compliance was scored over a 2-week (10-d) menu cycle period, with a maximum score for quantity and quality being fifty points each and a maximum score for variety being forty. For quantity, a maximum score of ten points was available, with one point allocated for each day that the recommended amounts of each of the five food groups (vegetables, fruit, dairy, meat/alternatives and grains) were provided. For quality, a maximum score of ten points was available, with one point allocated for each day that there was limited provision of discretionary items; refined grains; sweet and salty spreads and baked items at morning/afternoon tea (e.g. cakes, biscuits); saturated fats; and salt, For variety, a maximum score of ten points was available, with one point allocated for each day that there was provision of vegetarian meals, vegetable variety, fruit variety and meat variety. The maximum total menu score, indicating full compliance, was 140.

#### Barriers and enablers to menu compliance

Existing questions utilising the Theoretical Domains Framework (TDF) were used to assess barriers/enablers to dietary guideline implementation in LDC^(27,33,34)^. The TDF is a comprehensive, theory-informed approach for use in implementation research and behaviour change^(35)^ and has previously been adjusted to assess barriers and enablers to the implementation of menu planning guidelines in childcare settings^(27)^. A limitation identified by researchers of the previously adjusted TDF-based questionnaire in childcare settings^(27)^ was low internal consistency for some of the questions in certain TDF domains. This informed regrouping of questions in our study to more appropriately align to the childcare menu planning context. The TDF domains assessed in this study were as follows: (1) knowledge/awareness; (2) skills/role; (3) beliefs about capabilities/consequences; (4) optimism/intent; (5) reinforcement/influence; (6) environmental context and (7) behavioural regulation (see online Supplemental Table 2). Answer options included a five-point Likert scale ranging from *somewhat disagree (1)* to *strongly agree (5).* Items on the Likert scale were both positively and negatively phrased, and negative items were reverse-scored. Cronbach’s *α* was calculated for each TDF domain to assess internal consistency (range 0·7–0·9). Average scores for each TDF domain were calculated by the sum of items within the domain divided by the total number of questions in that domain. Mean average TDF domain scores were used to determine whether the domain was considered a potential barrier or enabler to menu planning^(27)^. Lower average scores (<4) were suggestive of the TDF domain being a barrier and higher average scores (≥4) that the TDF domain could be a potential enabler^(27,34)^.

### Statistical analysis

Summaries and tabulations were used for descriptive statistics. Independent *t* tests were used to assess differences in mean TDF scores and centre type, location, HEAS access and FoodChecker use. Chi-square tests were used to assess correlation between centre characteristics, support access and barriers/enablers to menu planning. One-way ANOVA assessed differences in the four aspects of menu scores by perceived barrier/enabler and by support service access. All data were analysed using statistical software STATA, version 16^(36)^.

## Results

### Characteristics of centres and persons responsible for menu planning

Of the 1442 listed centres, 138 provided consent and 89 completed the survey. Table 1 outlines the characteristics of participating centres. Most centres were in metropolitan areas (*n* 63) and privately operated (*n* 60). More centres were classified as high SEIFA (47 %) than medium (30 %) or low (23 %). Metropolitan and rural centres had a similar mean number of children attending (51 ± 30 and 56 ± 34, respectively). The survey was completed mainly by cooks (47 %) and centre director/ managers (40 %). Cooks mostly had sole responsibility for planning menus (92 %), purchasing (81 %), preparing (88 %) and cooking food (93 %). Budgeting was a shared responsibility for about half of centre director/managers (56 %). More than half (57 %) of the persons responsible for menu planning had not received any nutrition training to support this task. Of the thirty-nine persons who had received nutrition training, fifteen (38 %) had received training from HEAS, with remaining responses varied, including food allergy training (13 %) and a range of other peer learning situations.

**Table 1:** Descriptive statistics of centre characteristics and menu planning support (nutrition training, services and menu reviews)

	Total (*n* 89)		Metro total (*n* 63)		Rural total (*n* 26)		Community total (*n* 29)		Private total (*n* 60)	
Centre characteristic	*n*	%	*n*	%	*n*	%	*n*	%	*n*	%
SEIFA* low (≤3)	20	23	13	21	7	27	7	24	13	22
SEIFA medium (4–7)	27	30	19	30	8	31	8	28	19	32
SEIFA high (≥ 8)	42	47	31	49	11	42	14	48	28	47
Person planning menus received nutrition training										
Yes	39	43	25	40	14	54	17	59	22	37
No	50	57	38	60	12	46	12	41	38	63
Support access for menu planning										
Healthy Eating Advisory Service	50	55	33	52	17	65	16	55	34	57
Registered with the Achievement Program†	31	34	10	16	21	81	13	45	18	30
Other websites	22	25	50	79	19	73	25	86	44	73
Recipe websites	13	15	11	17	2	8	3	10	10	17
Missing data (website type unknown)	4	4	–		–		–		–	
Newsletters	17	19	13	21	4	15	5	17	12	20
Facebook	7	8	2	3	5	19	1	3	6	10
Menu reviewed										
Yes	50	57	33	52	17	65	21	72	29	48
No	39	43	30	48	9	35	8	28	31	52
Menu review details (n, % who provided data)	49	55	32	51	17	65	20	69	29	48
Reviewed by Centre staff (*n*, %)	14	29	10	31	4	23	6	30	8	28
Reviewed by Dietitian / Nutritionist (*n*, %)	5	10	2	6	3	18	2	10	3	10
Reviewed using Food Checker‡ (*n*, %)	30	61	20	63	10	59	12	60	18	62

* Socio-economic Indexes for Areas: Decile 1 = most disadvantaged, Decile 10 = least disadvantaged. Divided into low, medium and high categories^(25)^.

† The Achievement Program: Victorian Government funded program that recognises healthy early childhood services in six areas, one of them healthy eating and oral health^(27)^.

‡ Foodchecker: Menu planning tool for LDC provided through Healthy Eating Advisory Service (HEAS) – a Victorian Government funded nutrition support service for organisations including childcare^(22)^.

### Support for menu planning

About half the centres (55 %) had accessed HEAS for menu planning support, and 25 % accessed other websites, including recipe websites not specific to menu planning guidelines (15 %). Just over half of the centres (57 %) reported having had a menu review, most often utilising FoodChecker (61 %). More community-based centres had had their menu reviewed (72 %) than private centres (48 %) (*P* = 0·03). There was a statistically significant difference in FoodChecker usage between centres with staff who received nutrition training compared to those who had not (*P* < 0·01), with more nutrition-trained staff using FoodChecker. About a third of centres (34 %) were registered with the Achievement Program, a Victorian Government-funded program that recognises healthy early childhood services in six different areas, of which one, the healthy eating and oral health domain, requires compliant menus according to FoodChecker.

### Menu compliance

Eighteen LDC services provided a total of 180 daily menus, comprising 2 weeks of menu data and recipe details for each centre. The majority of centres who submitted menus were privately owned (*n* 12, 67 %) and located in metropolitan areas (*n* 14, 77 %), with 33 % (*n* 6) of centres being community-operated and 23 % located in rural areas (*n* 4). Sixty-one per cent (*n* 11) of centres who submitted menus were located in high SEIFA decile areas. Fifty per cent (*n* 9) had received nutrition training, and 67 % (*n* 12) had accessed HEAS for menu planning support. Fifty-six per cent (*n* 10) of centres had reviewed their menus in the past, and 44 % (*n* 8) had utilised FoodChecker for their menu review. As outlined in Table 2, one centre reached full menu compliance across all three menu score categories (quantity, quality and variety). This centre was also the only one to reach full compliance for quantity score. Lowest mean quantity scores were for grains (5·1 ± 3 points), vegetables (5·1 ± 3·2) and meat (5·4 ± 3·1). Fruit had the highest mean quantity score (8·1 ± 2·8). For quality scores, two centres reached full compliance. Lowest mean quality score was for wholegrains (5·2 ± 2·9). Half the centres reached full compliance for variety.

**Table 2:** Menu score results (total, quantity and quality, variety) – assessing menu compliance with the Victorian menu planning guidelines for long day care

Item	Mean score	sd	Score indicating full compliance	Number of centres achieving compliance with menu planning guidelines *n*	%
Total quantity score*	29·2	11	50	1	6
Mean score for vegetable provision	5·1	3·2	10	1	6
Mean score for fruit provision	8 .1	2·8	10	11	61
Mean score for dairy provision	5·6	3·5	10	5	28
Mean score for meat/alternatives provision	5·4	3·1	10	1	6
Mean score for grains provision	5·1	3	10	3	17
**Total quality score**	35·5	8·3	50	2	11
Discretionary items not on menu	8·7	2	10	9	50
Wholegrains on the menu daily	5·2	2·9	10	3	17
Sweet and salty spreads and baked items (cakes and biscuits) not offered daily	8·2	2·3	10	4	22
Mono- and polyunsaturated oils used in cooking, saturated fats not used, limit oils to 10 g/child/d	7	2·4	10	3	17
Salt not used in cooking or available on table, limit high salt sauces to 5 g/serve	6·5	2·6	10	3	17
**Total variety score†**	35·8	5·5	40	9	50
Vegetarian meals on menu (2/fortnight)	9·2	1·9	10	15	83
Vegetable variety (2 types/d, 5 types/week)	9·2	1·9	10	15	83
Fruit variety (2 types/d, 5 types/week	8·9	3·2	10	16	89
Meat variety	8·6	2·9	10	14	78
**Total menu score‡**	100·6	17·7	140	1	6

* Quantity and quality scores: based on 1 point for each day over a 2-week (10 d) period that each item was compliant (compliant = score of 50 each).

† Variety score: Relates to variety over a fortnight (10 d) – 5 points for each week compliant with each item (compliant = score of 40).

‡ Total menu score: Quantity + Quality + Variety (full compliance = score of 140).

### Barriers and enablers to menu planning

Of the average scores for each TDF domains (Table 3), knowledge/awareness and beliefs about capabilities/consequences scored highest (>4), indicating that these two domains are likely perceived as enablers to menu planning guideline implementation (*n* 89). All other domains had overall average scores <4, indicating that they are likely to be considered as barriers to menu planning guideline implementation. The TDF domains of reinforcement/influence and behavioural regulation had the lowest average scores. There were differences in optimism/intent average scores between metropolitan and rural centres (3·8 and 3·5, respectively) (*P* = 0·04). Those who accessed HEAS for support had higher average scores for the domains of knowledge/awareness, optimism/intent, skills/role and reinforcement/influence. Those who accessed FoodChecker had higher average scores for knowledge/awareness and skills/role. When analysed as proportion of centres viewing each domain as a barrier or enabler, there were two significant findings. Reinforcement/influence was more likely to be considered a barrier by privately operated centres (47 %) than community-operated centres (24 %) (*P* = 0·04), and skills/role was more likely to be considered a barrier by rural centres (46 %) than by metropolitan centres (19 %) (*P* = 0·01).

**Table 3:** Barriers and enablers (using Theoretical Domains Framework) to menu planning guideline implementation by centre type, location and support use. (A score of >4 indicates a perceived enabler and A score <4 indicates a perceived barrier)

Barriers/enablers	Average scores (maximum average score: 5)												
		Centre type			Centre location			Have used Healthy Eating Advisory Service			Have used FoodChecker		
	All	Private	Community	*P*-value	Metro	Regional	*P*-value	Access	No access	*P*-value	Usage	No usage	*P*-value
Reinforcement and influence (recognition and award/processes that that may influence guideline implementation)	3·3	3·2	3·4	0·07	3·4	3·2	0·1	3·4	3·2	0·05*	3·4	3·3	0·4
Behavioural regulation (self-monitoring and action planning for guideline implementation)	3·3	3·3	3·3	0·9	3·3	3·3	0·7	3·3	3·3	0·9	3·3	3·3	0·5
Skills and role (training skills and extent to which implementation of guidelines is perceived as being part of role)	3·6	3·6	3·7	0·9	3·7	3·4	0·1	3·8	3·4	0·03*	4·0	3·5	0·01*
Optimism and intent (confidence implementation is attainable and intention to implement guidelines)	3·7	3·7	3·8	0·4	3·8	3·5	0·04*	3·9	3·6	0·04*	3·9	3·7	0·2
Environmental context and resources (situation encourage/discourage implementation)	3·9	3·8	4·1	0·3	4·0	3·8	0·4	4·0	3·7	0·1	4·0	3·9	0·6
Capabilities and consequences (confidence in the implementation of and benefits of menu planning guidelines)	4·2	4·1	4·2	0·4	4·2	4·1	0·5	4·2	4·1	0·2	4·3	4·1	0·2
Knowledge and awareness (awareness of menu planning guidelines)	4·5	4·5	4·5	0·5	4·5	4·5	0·8	4·7	4·3	<0·01*	4·7	4·4	0·01*

* Statistically significant.

*P*-value of differences calculated using independent *t* tests.

### Association between government support access and menu score

A difference was found between menu quality scores for those accessing HEAS (38 ± 6·1 out of 50) and those not (30·2 ± 10·1 out of 50), (f: 4·5, *P* = 0·05), but there were no differences for quantity, variety or total menu scores.

### Association between barriers and enablers and menu score

There were no statistically significant associations between perceived barriers and enablers, and quantity, quality, variety or total menu scores.

## Discussion

The aim of this study was to describe adherence to menu planning guidelines, utilisation of support services, perceived barriers and enablers to menu planning, and any correlations between these in Victorian LDC services. We found that tasks related to menu planning, purchasing and preparing of food were mainly the responsibility of centre cooks, with the majority not receiving nutrition training to inform these roles, despite such training being freely available through HEAS. Of interest, we found that those who had received nutrition training were more likely to have reviewed their menus using the menu planning tool, FoodChecker. Whilst a relatively low number of centres responded to our survey, which may limit the generalisability of the findings, our findings endorse previous research indicating the pivotal role of childcare cooks and both the importance^(37)^ yet low levels of^(14,38)^ nutrition training for these staff.

Childcare-specific government-funded resources provide one example of supporting the upskilling of childcare cooks. Little research has been conducted on accessing government resources and/or training for childcare menu planning in Australia. Our study found a moderate uptake of the Victorian Government-funded support service HEAS, with just over half of centres participating in our study accessing this service. Given the small sample size of our study, larger studies are recommended to confirm these findings. Further, although HEAS is a publicly available, free service, it is important to understand barriers to accessing such a service to ensure broad reach and utilisation. A recent Australian qualitative study^(37)^ conducted in South Australia found most cooks were disinterested and expressed difficulties in accessing online government support and preferred face-to-face training^(37)^. South Australia has no state government-funded service to support menu planning in childcare and centres use the nationally available ‘Get up and Grow’ resources^(39)^. This national resource provides a suite of information on early childhood nutrition and childcare-specific recommendations but no easily accessible information on serving sizes for childcare food provision, no tools to assess menu compliance against guidelines, and no training to assist with resource interpretation and implementation. Cooks in the South Australian study expressed a preference for face-to-face training with ongoing online support, consistent with Victoria’s HEAS support which offers online and face-to-face training, online resources, and telephone support. Whilst HEAS provides training in a scalable manner across Victoria and some other Australian states, investigating the reach of this support, and how it might be enhanced, is important to consider in future research.

Most centres considered ‘knowledge/awareness’ and ‘capabilities/consequences’ as enablers to menu planning, with ‘environment/resources’, ‘optimism/intent’, ‘skills/role’, ‘behavioural regulation’ and ‘reinforcement/influence’ viewed as barriers. Consistent with this, a recent study conducted in New South Wales (NSW), Australia, which used the TDF to assess barriers to dietary guideline compliance, reported ‘knowledge’ and ‘capabilities’ as enablers, and ‘reinforcement’ and ’behavioural regulation’ as barriers to menu planning^(27)^. In contrast to the NSW study, who reported ‘optimism/intent’, ‘skills/role’ and ‘environmental context and resources’ as enablers, our study found these to be considered barriers. One explanation for these differences may be explained by the internal consistency of the survey questions, with the NSW study reporting a Cronbach’s *α* of < 0 70 for the domains ‘optimism’ (0·67) and ‘skills’ (0·61) and recommending improvements be made for these domain questions. This was considered for our study resulting in greater internal consistency >0·70 of survey questions (0·71 and 0·79 for ‘optimism/intent’ and ‘skills/role’, respectively). Another, and perhaps more important explanation, is that NSW also has a government-funded childcare-specific support service known as ‘Munch & Move’^(40)^, which provides training and resources to LDC. A 2015 report indicated that 89·8 % of LDC staff in NSW had received training through a workshop provided by ‘Munch & Move’^(41)^, compared to less than half who had received nutrition training in our study. This increased uptake of government-funded training could explain why most TDF domains were viewed as enablers in the NSW study, compared to the majority being considered barriers in our study. This emphasises the importance of understanding the uptake (or lack thereof) of publicly available, free services and reasons underlying this.

Our study indicated that those who accessed HEAS and used the HEAS menu planning tool, FoodChecker, had higher scores for ‘knowledge/awareness’, ‘optimism/intent’ and ‘skills/role’. Whilst direction of influence cannot be determined in a cross-sectional study, this may indicate that these participants felt supported in all these TDF domains. These findings may suggest that providing support to translate dietary guidelines into childcare-specific menu planning guidelines is not only associated with higher knowledge and skills as intended but also with staff perceptions that implementation is attainable. Our study also found that ‘reinforcement/influence’ scored lowest overall. Previous research has reported that the ‘reinforcement’ domain, which relates to recognition and award for implementing guidelines, is strongly influenced by centre directors who are responsible for the processes that reward and recognise staff for following guidelines^(42)^. Whilst our study focused on the importance of targeted support for centre cooks, this may indicate that support should also be provided to centre directors, particularly in relation to creating and maintaining environments that positively reinforce menu compliance.

We found that access to HEAS and its association with improved menu quality approached significance (*P* = 0·05) and warrants further research with a larger sample. To our knowledge, this is the first Australian study to score food provision in childcare in terms of quantity, quality and variety, and to find that access to government-funded childcare-specific support may be associated with higher menu quality. To confirm this, further research with a study design to establish causation is recommended.

In comparison with the previously mentioned NSW study, in which ‘skills’ was the only TDF domain found to be associated with improved menu compliance^(27)^, our study found that ‘skills/role’ scores were higher for those who accessed HEAS and FoodChecker; however, there were no significant associations with menu compliance. An important difference between the studies was the measurement of compliance, which only focused on quantity of foods served in the NSW study, without measurement of quality and variety. These findings may imply that despite government-funded childcare-specific support being accessed by about half of the centres in our study, and being associated with improved perceived knowledge, skills and optimism in centre staff, this does not necessarily translate into menu compliance. This was evident in our study with only one of eighteen menus being compliant. These findings are congruent with those reported in a recent Cochrane review assessing strategies to improve implementation of healthy eating and obesity prevention policies and programmes within childcare services^(43)^, which found that whilst training of staff led to improved centre-based healthy eating policy, this infrequently translated into menus compliant with dietary guidelines^(43)^.

One plausible explanation for this may be that government-funded support and resources predominantly address knowledge, confidence and skills, with less focus on factors influencing reinforcement and the environmental context to achieving compliant menus. Contextual barriers identified in other studies include perceptions regarding the cost of and food wastage associated with healthier menus and children’s food preferences, which may influence menu planning and food provision decisions^(13,44,45)^. Incorporating guidance on these factors within existing government-funded support is considered important to address these barriers to menu planning implementation.

Importantly, the apparent limited application of support into menu compliance by childcare services, as found in our study and previous research, may also be explained by how menu compliance is assessed. Assessing menus against overall compliance does not allow for the detection of any incremental improvements that could be made. For example, our study found that access to HEAS may be associated with higher menu quality scores. Whilst this does not equate to full menu compliance, and the findings should be considered in the light of the study design and sample size, improving scores across the menu components may be a step in the right direction from a public health perspective and could inform potentially more tailored strategies to improve menu compliance. This has also been suggested in an Australian intervention study which found the use of a web-based menu planning tool improved food provision, but full menu compliance was not reached in any of the participating centres^(46)^. Full menu compliance appears complex and may be largely unattainable in conjunction with managing food wastage and budget^(16,42)^. Larger studies are needed to explore this. In addition, future research into the assessment of menu compliance components, the tailoring of strategies to achieve these components and perceptions of whether menu compliance is reasonable and attainable may provide a more realistic approach to achieving healthy childcare menus.

Of interest in the current study was the finding that, compared to community-managed services, privately owned LDC services were reportedly less likely to have nutrition-trained staff responsible for menu planning, less likely to have their menu reviewed and less likely to be rewarded or recognised for planning complaint menus. Similarly, a New Zealand study found that community-operated centres had higher menu scores compared to privately operated centres^(11)^. Whilst our study did not consistently find differences in barriers or enablers between privately owned *v*. community-managed services or those located in rural and metropolitan areas, the TDF domains ‘skills/role’ and ‘optimism/intent’ did have lower average scores in rural LDC services compared to metropolitan. A recent study reporting differences in menu compliance by centre type and location^(11)^ indicates a need for further research, in larger samples, to explore the potential for tailored support and resources by centre type and location.

Our study has several limitations. As a cross-sectional study, no causality can be inferred. The modest number of participants, and small number of services providing menus has implications for generalisability, with adjustment for known confounders not possible. Studies with larger sample sizes are therefore recommended to confirm findings. Participant burden to provide menus and recipes for a 2-week period may have posed a barrier, limiting the ability to assess full menu compliance against the Victorian menu planning guidelines. Further research on the use of menu review tools such as FoodChecker and how to improve ease of use can inform this. Whilst study participants may represent centres with a greater interest in food provision and higher SES, the low level of menu compliance across the sample would suggest that results may depict usual practice, highlighting that further assessment of food provision in diverse centres is a public health priority. Our study only assessed barriers and enablers within the influence of an individual responsible for menu planning and did not explore organisational factors such as food costs, children’s food preferences and food wastage. Future research exploring strategies to overcome these potential barriers is important to enhance menu compliance and support the development of healthy food preferences in children, whilst also addressing the public health concern of environmental sustainability^(47)^.

## Conclusions

To the authors’ knowledge, this is the first quantitative study to assess the uptake and usage of a government-funded childcare-specific support agency and correlations with perceived barriers and enablers to childcare menu planning guideline implementation. Our findings suggest that childcare-specific menu planning resources and support could be an important public health strategy, associated with higher perceived knowledge, confidence and skills for menu planning as well as optimism that menu compliance is attainable. Furthermore, uptake and usage of current support services may be associated with improved menu quality; however, this does not necessarily translate into menu compliance. Further research, with larger studies, should assess the uptake and acceptance of government-funded support. Public health efforts should also be directed to increasing the promotion, awareness and uptake of these available resources and support, targeting all centre staff including cooks and directors, addressing contextual factors and organisational barriers to reinforcement. Finally, exploring menu compliance in terms of its components of quantity, quality and variety may provide a more discerning method to review childcare menus rather than simply assessing compliance and enable the design of tailored strategies to improve menu planning guideline implementation and healthy childcare menus.

## Supplementary material

For supplementary material accompanying this paper visit https://doi.org/10.1017/S1368980022001343. click here to view supplementary material
