# Supplementary material for: Barriers and enablers to menu planning guideline implementation in Australian childcare centres and the role of government support services
Source: Public Health Nutr. 2022 Jun 1;25(10):2661–70. doi: 10.1017/S1368980022001343 (PMC9991762; doi:10.1017/S1368980022001343)
Supplement: Supplementary file 1 [file S1368980022001343sup.zip › S1368980022001343sup001.docx]

| **Quantity**  **(1 point per day of full compliance for each food group (10 points over the fortnight))** | **Quality**  **(1 point per day compliant for each item (10 points over the fortnight))** | **Variety**  **(5 points for each week each component is compliant (10 points over the fortnight))** |
| --- | --- | --- |
| **Meat/alternative compliant**  (Offer 1 child serve per child per day – 1 child serve equivalent to: 30g cooked lean meat / 1 egg / 85g legumes) | **Discretionary not on menu.**  (Discretionary foods include chocolate, jelly, commercially made biscuits, ice cream, soft drinks, fruit juice, deep fried foods and take away foods) | **Vegetarian meals**  (2 per fortnight – a vegetarian meal should contain protein such as legumes, food containing iron e.g., spinach or chickpeas, vegetables high in vitamin C) |
| **Vegetables compliant**  (Offer 1-1.5 child serve per child per day – 1 child serve equivalent to: ½ cup cooked vegetables / 1 cup salad / ½ potato) | **Wholegrains on the menu every day**  (Includes oats, brown rice, rice, and wholemeal varieties for breads and in baking) | **Vegetable variety**  (2-3 different types per day and 5 different types each week) |
| **Fruit compliant**  (Offer 1 child serve per child per day - 1 child serve equivalent to: 75g fresh fruit) | **Sweet and salty spreads and baked items not offered daily at morning or afternoon tea. Contains <5g sugar / serve.**  (Sweet spreads include jam and honey. Salty spreads include Vegemite™ and Marmite™. Baked items should contain less than 5g of sugar per serve and should not be offered daily at morning or afternoon tea) | **Fruit variety**  (2-3 different types per day and 5 different types each week) |
| **Milk/alternatives compliant**  (Offer 2 child serves per child per day – 1 child serve equivalent to: 100ml milk/ 80g yoghurt / 15g hard cheese) | **Mono- and poly unsaturated oils used in cooking, saturated fats not used in cooking, limit oils to 10g / child / day.**  (Suitable options include olive oil, canola oil, sunflower oil – avoid palm and coconut oil as well as cream, butter, and lard) | **Meat / protein variety**  (Over a fortnight – offer 4 serves of red meat, 2 serves of poultry, 2 serves of fish and 2 vegetarian meals) |
| **Grains compliant**  (Offer 2 child serves per child per day – 1 child serve equivalent to: 1 slice bread / 30g dry weight pasta or rice / 30 crisp bread or crackers) | **Salt not used in cooking or available on table, limit high salt sauces to 5g / serve.**  (Sauces such as soy sauce, fish sauce and tomato sauce can be high in salt – choose reduced salt options and use in small amounts) |  |
| **Compliance quantity = 50** | **Compliance quality = 50** | **Compliance variety = 40** |
| **Full compliance = 140*** | | |

**Supplementary Table 1 – Menu scoring tool based on the Victorian menu planning guidelines for Long Day Care.**

*Items in menu scoring system derived from methodology created by Dean ^(32)^, utilised by Gerritsen ^(11)^ and adjusted to the recommendations in the Victorian menu planning guidelines for Long Day Care ^(19)^ in collaboration with Nutrition Australia’s Healthy Eating Advisory Service.^(48)^
