# Supplementary material for: Barriers and enablers to menu planning guideline implementation in Australian childcare centres and the role of government support services
Source: Public Health Nutr. 2022 Jun 1;25(10):2661–70. doi: 10.1017/S1368980022001343 (PMC9991762; doi:10.1017/S1368980022001343)
Supplement: Supplementary file 1 [file S1368980022001343sup.zip › S1368980022001343sup002.docx]

**Supplementary Table 2: Survey items pertaining to barriers / enablers to menu planning based on the Theoretical Domains Framework**

| **Knowledge & Awareness** |
| --- |
| I am aware of the menu planning guidelines* |
| I understand why it is important to apply the menu planning guidelines |
| I know how to plan a menu according to the menu planning guidelines |
| The menu planning guidelines ensure children have the right amounts of dairy, meats, fruits, vegetables and grains each day while in care |
| The menu planning guidelines ensure children have healthy meals and snacks while in care |
| **Skills & Role** |
| I have received training regarding how to plan a menu according to the menu planning guidelines |
| I have the skills to plan a menu according to the menu planning guidelines |
| Planning a menu according to the menu planning guidelines is part of my role |
| Compared to my other tasks, planning a menu according to the menu planning guidelines is a higher priority |
| **Capabilities & Consequences** |
| I am confident that I can plan a menu according to the menu planning guidelines |
| I believe that planning a menu according to the menu planning guidelines will provide benefits for the children in my Centre |
| In my view, planning a menu according to the menu planning guidelines is useful |
| For me, planning a menu according to the menu planning guidelines is easy |
| **Optimism & Intent** |
| I do not expect anything to prevent me from planning a menu according to the menu planning guidelines |
| Planning a menu according to the menu planning guidelines is something I do automatically |
| Planning a menu according to the menu planning guidelines is something I forget (reverse scored) |
| I am able to plan a menu according to the menu planning guidelines without feeling anxious |
| I am able to plan a menu according to the menu planning guidelines even when I feel stressed |
| **Reinforcement & Influence** |
| I receive recognition from my workplace when I plan a menu according to the menu planning guidelines |
| I intend to plan a menu according to the menu planning guidelines every time |
| I intend to plan a menu according to the menu planning guidelines in the next 6 months |
| People whose opinion I value would approve of me planning a menu according to the menu planning guidelines |
| **Environmental context & resources** |
| In my workplace, I have all the necessary resources to plan a menu according to the menu planning guidelines |
| In my workplace, I have support from management to plan a menu according to the menu planning guidelines |
| In my workplace, management are willing to listen to any problems I have when planning a menu according to the menu planning guidelines |
| In my workplace, I have the opportunity for training to plan a menu according to the menu planning guidelines |
| In my workplace, I have sufficient time to plan a menu according to the menu planning guidelines |
| In my workplace, I have sufficient financial support for me to plan a menu according to the menu planning guidelines |
| In my workplace, I have the support of colleagues when planning a menu according to the menu planning guidelines |
| **Behavioural regulation** |
| I follow a specific process when planning a menu to meet the menu planning guidelines |
| I am able to make changes to the menu if children don't like the food and still meet the menu planning guidelines |
| When I plan a menu I include foods that I believe are healthy for the children |
| When I plan a menu I include foods that I know the children will prefer (reverse scored) |
| When I plan a menu I include foods that I know will fit with the budget |
| When I plan a menu I choose foods that I like (reverse scored) |
| When I plan a menu I choose foods that are easy to prepare and/or cook (reverse scored) |

*Menu Planning guidelines refer to the Victorian menu planning guidelines for Long Day Care

Questions were answered on a 5-point Likert-scale ranging from strongly agree to strongly disagree
